# Leaf thermotolerance in dry tropical forest tree species: relationships with leaf traits and effects of drought

**DOI:** 10.1093/aobpla/plx070

**Published:** 2017-12-11

**Authors:** Aniruddh Sastry, Anirban Guha, Deepak Barua

**Affiliations:** Department of Biology, Indian Institute of Science Education and Research, Pune, India

**Keywords:** Climate change, drought, leaf functional traits, photosynthesis, PSII chlorophyll fluorescence, thermotolerance, tropical forests

## Abstract

Understanding how tropical trees will respond to extreme temperatures and drought is essential to predict how future increases in the severity, frequency and duration of extreme climatic events will affect tropical systems. In this study, we investigated leaf thermotolerance by quantifying the temperatures that resulted in a 50 % decrease in photosystem II function (T_50_) in experimentally grown saplings of 12 tree species from a seasonally dry tropical forest. We examined the relationship of thermotolerance with leaf functional traits and photosynthetic rates. Additionally, we tested how water limitation altered thermotolerance within species, and examined the relationship between thermotolerance and drought tolerance among species. Thermotolerance ranged from 44.5 to 48.1 °C in the least and most thermotolerant species, respectively. The observed variation in thermotolerance indicates that the upper limits of leaf function are critically close to maximum temperatures in this region, and that these species will be vulnerable to, and differentially affected by, future warming. Drought increased temperature tolerance, and species that were more drought tolerant were also more thermotolerant. Importantly, thermotolerance was positively related to the key leaf functional trait—leaf mass per area (LMA), and congruent with this, negatively related to photosynthetic rates. These results indicate that more productive species with lower LMA and higher photosynthetic rates may be more vulnerable to heat and drought stress, and more likely to be negatively affected by future increases in extreme climatic events.

## Teaser

We observed that the upper thermal limits of leaf function in 12 tree species from a seasonally dry tropical forest were perilously close to maximum temperatures in this region. Exposure to drought increased thermotolerance, and species that were more drought tolerant were also more thermotolerant. Importantly, thermotolerance was positively related to the key leaf functional trait - leaf mass per area, and negatively related to photosynthetic rates. These results indicate that tropical trees will be vulnerable to increased warming, and more productive species with lower LMA and higher photosynthetic rates may be more negatively affected by global warming and future climate change.

## Introduction

With future global warming, plants will experience extreme temperatures more frequently (Meehl and Tebaldi 2004; Hansen *et al.* 2012), and this will often be accompanied by reduced water availability and drought (Toomey *et al.* 2011; Niu *et al.* 2014; Allen *et al.* 2015). Tropical species may be particularly vulnerable to future warming as they are already living closer to their absolute thermal limits, have narrower thermal niche breadth and may be limited in their ability to acclimate to warmer temperatures (Deutsch *et al.* 2008; Doughty and Goulden 2008; Cheesman and Winter 2013; O’Sullivan *et al.* 2017). In dry tropical forests this vulnerability is likely to be exacerbated by exposure to more severe and frequent drought (Allen *et al.* 2017). Such exposure to extreme climatic events will increase cellular stress and damage, which in turn will decrease photosynthesis and growth, and could ultimately result in mortality of individuals (Niu *et al.* 2014; Teskey *et al.* 2015). Knowledge of species upper limits of physiological tolerance to high temperatures, and how these limits are affected by reduced water availability, is important to understand the vulnerability of tropical trees, and predict responses of plants and communities, to future climate change.

A recent study demonstrated that the upper thermal limits of leaf photosynthetic and respiratory function in over 200 tree species were inversely related to latitude (O’Sullivan *et al.* 2017). This confirmed previous results that suggested higher thermotolerance in tropical plants compared to temperate and alpine plants (Lange and Lange 1959; Weng and Lai 2005; Cunningham and Read 2006). However, in contrast to the 20 °C difference in maximum habitat temperatures between sites, the increase in average thermotolerance from the poles to the tropics was only ~8 °C (O’Sullivan *et al.* 2017). This means that the upper limits of tolerance in tropical plants are closer to the maximum habitat temperatures that they experience. Additionally, these results highlight the large variation in thermotolerance observed between species within sites. As previously reported, variation in thermotolerance in coexisting species can range from 10 to 20 °C (Lösch 1980; Gauslaa 1984; Knight and Ackerly 2003; Weng and Lai 2005; Neuner and Buchner 2012).

The shallow relationship between habitat temperatures and thermotolerance, and the large variation within coexisting species, suggests a decoupling between habitat and leaf temperature. Differences between habitat temperatures and leaf temperatures can result from fine-scale spatial heterogeneity in light, wind speed, water availability and temperature (Gauslaa 1984; Curtis *et al.* 2016), but can also result directly from variation in leaf traits (Curtis *et al.* 2012; Leigh *et al.* 2017). Leaf structural and morphological traits affect the relationship between leaf and air temperatures, and can determine the range and extremes of temperatures experienced (Grace *et al.* 1980; Leigh *et al.* 2017). The ability of leaves to regulate temperature may be more extensive than previously believed (Helliker and Richter 2008; Song *et al.* 2011), and it has been suggested that key leaf traits like leaf mass per area (LMA) and leaf dry matter content (LDMC) may be fundamentally interlinked to leaf temperatures and carbon economies (Michaletz *et al.* 2016; Slot *et al.* 2017).

Curtis *et al.* (2012) proposed that LMA should be positively related to thermotolerance based on the covariation between LMA and other leaf traits that are important for thermal protection, e.g. leaf size and thickness, pendulousness and spectral properties. A relationship between key leaf functional traits like LMA and thermotolerance would suggest that species vulnerability to extreme temperature might vary with position along the slow-fast resource acquisition trade-off axes (Wright *et al.* 2004; Reich 2014). Species that maximize resource acquisition with low LMA, high photosynthetic rates and short leaf lifespans might be expected to have lower thermotolerance, and conversely, those that maximize leaf longevity with high LMA, low photosynthetic rates and long lifespans would have high thermotolerance. Understanding such a relationship would give us predictive insights into responses of species and communities to future global warming, such as identifying vulnerable species, predicting future changes in community composition and understanding the consequences of such changes in terms of ecosystem properties and function.

Only a handful of studies have examined the relationship between thermotolerance and leaf functional traits associated with the leaf economic spectrum, and the results from these are equivocal with some demonstrating positive relationships (Knight and Ackerly 2003; Gallagher 2014; Sastry and Barua 2017), while others have failed to detect any covariation between leaf functional traits and thermotolerance (Zhang *et al.* 2012; O’Sullivan *et al.* 2017). However, previous studies were conducted on naturally occurring plants where microhabitat conditions of individuals were not controlled, and could confound results observed. Additionally, the relationship to resource acquisition strategies is indirect as these previous studies did not measure photosynthetic rates.

Extreme temperatures are often accompanied by low soil water availability and increased vapour pressure deficits (Stéfanon *et al.* 2014), and this could further exacerbate heat-induced damage by limiting transpirational cooling. Both heat and drought cause increased oxidative stress and damage at the cellular level, and result in the induction of similar protective mechanisms (Wang *et al.* 2003). Likely as a consequence of such common responses, exposure to drought increases the ability to withstand other abiotic stress including high temperatures (Ladjal *et al.* 2000; Gauthier *et al.* 2014). A previous study reported that drought tolerance is positively related to thermotolerance in 45 varieties of cereals (Havaux *et al.* 1988), but whether this is true for other plants, particularly in dry tropical forest trees that are adapted to hot and dry environments, is not known.

In this study, we quantified leaf thermotolerance in saplings of 12 seasonally dry tropical forest tree species that were grown in a common environment. We measured the temperature response of dark-adapted chlorophyll *a* fluorescence (*F*_*v     *_*/F*_*m*_), and estimated the temperature that results in a 50 % loss of function (T_50_). The temperature response of chlorophyll *a* fluorescence is a physiological measure of the integrity of the thylakoid membrane, is highly sensitive to high temperature, represents a good indicator of photosynthetic and organismal thermotolerance (Björkman and Demmig 1987; Havaux *et al.* 1991; Barua *et al.* 2003) and has been used extensively in determining plant sensitivity to extreme temperatures (Cunningham and Read 2006; Barua *et al.* 2008; Zhang *et al.* 2012; Curtis *et al.* 2014; O’Sullivan *et al.* 2017). Specifically, we examined the relationships between thermotolerance and leaf functional traits—LMA, LDMC, leaf size and photosynthetic rates. Additionally, we examined how exposure to experimental drought affects photosystem II (PSII) function at high temperature, and tested whether this was positively related to drought tolerance. Performance under drought conditions was quantified by measuring leaf wilting, leaf relative water content (RWC) and the decrease in photosynthesis as compared to well-watered plants (Engelbrecht *et al.* 2007; Saura-Mas and Lloret 2007). These measures provide valuable information about leaf water status in response to drought, and were used to rank the relative drought tolerance of the study species.

## Methods

### Species selection and growth conditions

We selected 12 tree species that are commonly found in the seasonally dry forests of the Northern Western Ghats of peninsular India **[see Supporting Information—Table S1]**. The vegetation in this region varies from scrub/savanna to semi-evergreen forests. The climate is seasonal and most of the annual rainfall of ~2000 mm falls between June and September **[see Supporting Information—Fig. S1]**. Monthly minimum temperatures in January average 11 °C while maximum temperatures in April average 37 °C. Ten of the 12 species were selected from 80 species for which leaf functional trait and ~3 years of leaf phenology were available (D. Barua, unpubl. data). This allowed us to identify and select representative species that span the range of leafing behaviour and leaf functional trait values observed in this region **[see Supporting Information—Fig. S2]**.

The study was conducted between May and July 2015 at the Indian Institute of Science Education and Research (IISER) campus, Pune, India. For all species, 12 individuals of 2.5-year-old saplings [**see Supporting Information—Table S2** for details of height and stem diameter] were obtained from a local nursery (J.E. Farms, Pune), and transplanted to 19 L PVC pots (60 cm length, and 20 cm diameter) filled with 18 kg dry red alfisol (pH 7.2) supplemented with organic manure (1:50 v/v). Saplings were moved to a greenhouse and given a period of 6 weeks to acclimate before the experiment. All plants were fertilized once, 15 days after transplantation, with urea (0.05 g·kg^−1^ soil). The greenhouse received natural sunlight supplemented with incandescent lamps to ensure photosynthetic photon flux density (PPFD) of 500–800 µmol·m^−2^·s^−1^ (between 0900 and 1600 h). Mean daily temperature in the greenhouse ranged between 25 and 31 °C, while relative humidity (30–55 %) varied according to local conditions.

### Drought treatments

At the start of the experiment, the 12 individuals of each species were randomly assigned to the two treatments (6 control—well watered; 6 drought) and randomly allocated positions in the greenhouse. Before the beginning of the treatments, all pots were fully saturated with water in the evening, excess water allowed to drain overnight and weighed the next morning to determine the pot weight at field capacity. A white plastic sheet was taped to the pot rim and loosely tied around the base of the plants to minimize evaporation from the soil. During the experiment all control pots were individually weighed every 3 days, the loss of water quantified and the appropriate volume of water added to bring the pot back to 90 % of its field capacity. In this manner all control plants were always maintained at 75–90 % field capacity.

Drought was imposed by termination of watering at the start of the experiment. These pots were weighed every 3 days to estimate water loss and determine the point at which the pot water reached 30 % of field capacity. To standardize the drought treatment across these species that varied widely in their water use, we used the time at which pots reached 30 % of field capacity as the end point of the drought treatment. This pot water content was chosen to ensure that all species showed significant signs of water limitation, but also so that the drought treatment did not result in severe leaf necrosis and death in any of the species. The affect of drought was not examined for *Garcinia indica* because of the lack of sufficient plants. At the end of the drought treatment we measured gas exchange, quantified leaf wilting and collected leaf samples for thermotolerance assays and estimation of leaf RWC.

### Estimation of leaf wilting stage and RWC

Average leaf wilting stage scores were estimated and RWC quantified in the morning after drought-stressed plants reached 30 % of field capacity. Average leaf wilting was scored in a semi-quantitative manner (Engelbrecht *et al.* 2007) for the third, fourth and fifth leaves from the apex for 5–6 individuals per species. Wilting was assessed as change in leaf angle relative to the stem axis as compared to control leaves, by rolling and folding of leaves, and necrosis and chlorosis, and scored from 1 to 5 based on categories defined by Engelbrecht *et al.* (2007). Briefly, stage 1—no signs of wilting or damage; stage 2—slight change in leaf angle, but no rolling or folding; stage 3—pronounced change in leaf angle or protrusion of veins; stage 4—extreme change in leaf angle with beginning of cell death; 5—complete necrosis of the leaf.

For quantification of leaf RWC, leaf discs (1 cm^2^) were excised with a cork borer from the middle of the first fully expanded mature leaf taking care to exclude the midvein. The discs were weighed to quantify fresh weight (FW), water saturated for 24 h at 4 °C and subsequently the turgid fresh weight (TW) measured. Leaf discs were then put in a drying oven at 70 °C for 3–4 days till a constant dry weight (DW) was obtained. Leaf RWC (Saura-Mas and Lloret 2007) was calculated as: RWC = 100 × (FW − DW)/(TW − DW) for six individuals of each species.

### Gas exchange measurements

Leaf gas exchange was measured for the first fully expanded leaf for six individuals each for control (well watered) and drought-stressed plants (at the end of the drought treatment) with a LI-6400XT portable photosynthesis system (LI-COR, Lincoln, NE, USA) using the standard broadleaf cuvette (6 cm^2^) fitted with the LICOR-6400-02B LED light source. These measurements were made between 0930 and 1130 h with the cuvette light, CO_2_ concentrations (incoming reference), relative humidity and temperature set at 800 μmol m^2^ s^−1^ PPFD, 390 ± 10 ppm, 50–60 % and 28–30 °C, respectively.

### Quantification of leaf functional traits

LMA (g m^−2^), LDMC (mg g^−1^) and leaf area (LA, cm^2^) were quantified for five fully expanded and mature leaves from six individuals of the control (well watered) plants as per protocols recommended by Pérez-Harguindeguy *et al.* (2013). Leaves were water saturated for 12 h at 4 °C, and the saturated fresh weight obtained. They were then scanned with a desktop scanner to quantify LA, and put in a drying oven at 70 °C for 3–4 days till a constant dry weight was obtained. Leaf mass per area was quantified as the ratio of dry weight to one-sided leaf surface area, and LDMC as the ratio of dry weight and saturated fresh weight.

### Temperature tolerance assays

For control (well watered) plants, we measured the temperature response of dark-adapted chlorophyll *a* fluorescence (*F*_*v     *_*/F*_*m*_), and estimated the temperature that results in the 50 % loss of function (T_50_). *F*_*v*_*   /F*_*m*_ represents the maximum potential quantum yield of PSII and was calculated as *F*_*v  *_* /F*_*m*_ = (*F*_*m*_ − *F*_*o*_)/*F*_*m*_, where *F*_*m*_ and *F*_*o*_ are the maximum and basal fluorescence yield, respectively, for dark-adapted leaves. Leaf discs (2 cm^2^) were placed between two layers of muslin cloth, covered with aluminium foil and put in a sealed plastic lock bag. A moistened wad of tissue paper was put in the bag to maintain high water vapour content and prevent dehydration in the leaf. The plastic bag was immersed in a temperature-controlled refrigerated water bath (Julabo, Model F25, Seelbach, Germany) set to achieve the desired leaf temperature (25, 40, 45, 47.5, 50 °C) for 30 min. Separate leaf discs from independent leaves from the same individuals were used for each temperature. Preliminary trials and previous studies (Curtis *et al.* 2014) showed that a 30-min exposure resulted in irreversible damage and negligible recovery. The temperature of dummy leaf discs (not used for assays) was monitored with a thermocouple attached to the underside of the disc. Preliminary trials were conducted to determine the temperature of the water bath required to maintain the desired leaf temperatures. Following the temperature treatment, leaf discs were allowed to dark adapt for 30 min in the dark in a water-saturated environment at room temperature before measurement of *F*_*v*_*   /F*_*m*_ using a PAM 2500 fluorometer (Walz, Effeltrich, Germany).

A four-parameter logistic sigmoid curve was fitted to the chlorophyll *a* fluorescence (*F*_*v*_*   /F*_*m*_) values across the range of temperatures examined using the R package ‘drc’ (Ritz and Streibig 2005). The four-parameter model with the lower asymptote set to zero was observed to generate appropriate curves. The temperature (T_50_) at which reduction in *F*_*v      *_*/F*_*m*_ was 50 % of the upper asymptote was estimated from these curves. We used five independent leaf discs from an individual at each of the temperatures to generate an *F*_*v   *_*  /F*_*m*_ response curve from which we estimated T_50_ for that individual. This was repeated for 5–6 individuals for each species. For the drought-stressed plants we measured *F*_*v*_*   /F*_*m*_ at 25, 47.5, 50 °C due to limited availability of leaf samples, and thus did not calculate T_50_. Based on knowledge that variation between species is maximal at 47.5 °C, we use PSII function at this temperature as an index of thermotolerance that reflects the relative ranking of species performance at high temperatures.

### Statistical analyses

We examined variation in T_50_ of PSII function for the control plants using a one-way ANOVA with species as a fixed effect. To test how experimentally imposed drought affects thermotolerance in these species, we examined variation in *F*_*v   *_*/F*_*m*_ using an ANOVA with species, treatment (control and drought) and temperature (25, 47.5, 50 °C) as fixed effects. Variation in LMA, LDMC, LA, wilting score and RWC was examined using ANOVA with species as a fixed effect. We performed non-parametric Kruskal–Wallis tests for LMA, LA and wilting scores as these were not normally distributed. Variation in photosynthetic rates was examined with a two-way ANOVA with species and treatment (control and drought) as fixed effects.

We used Pearson’s correlations to examine relationships between thermotolerance (T_50_ of PSII for control, and *F*_*v*_*   /F*_*m*_ at 47.5 °C for drought treatments), leaf traits (LMA, LDMC, LA and photosynthetic rates) and drought tolerance (leaf RWC, wilting score, change in photosynthetic rates with drought). Additionally, we examined Spearman’s rank correlations for analyses with LMA, LA and wilting scores as these variables were not normally distributed. All analyses were performed using Statistica (version 9.1, Statsoft, Tulsa, OK, USA).

## Results

**Figure 1.**
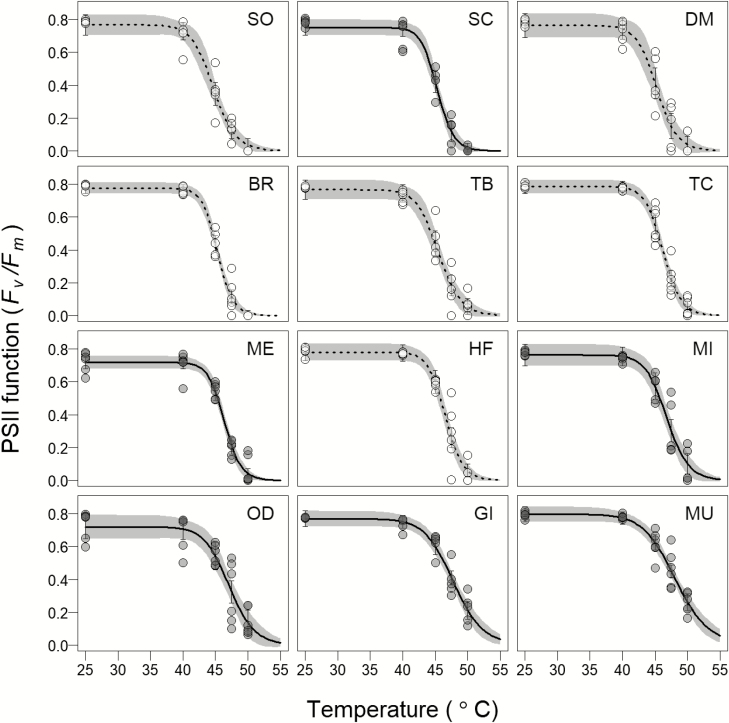
Temperature response of PSII function (dark-adapted chlorophyll *a* fluorescence - *F*_*v*_   /*F*_*m*_) in control (well watered) plants of the 12 tree species examined. The open and closed gray circles represent the *F*_*v*_   /*F*_*m*_ values for deciduous and evergreen species, and the dashed and the solid lines represent a logistic sigmoid fit for deciduous and evergreen species, respectively. Error bars and the shaded portion indicate the 95 % CI (*n* = 5–6 individuals for each species). Species are arranged in increasing order of thermotolerance. Species names are provided in **Supporting Information—Table S1**.

**Figure 2.**
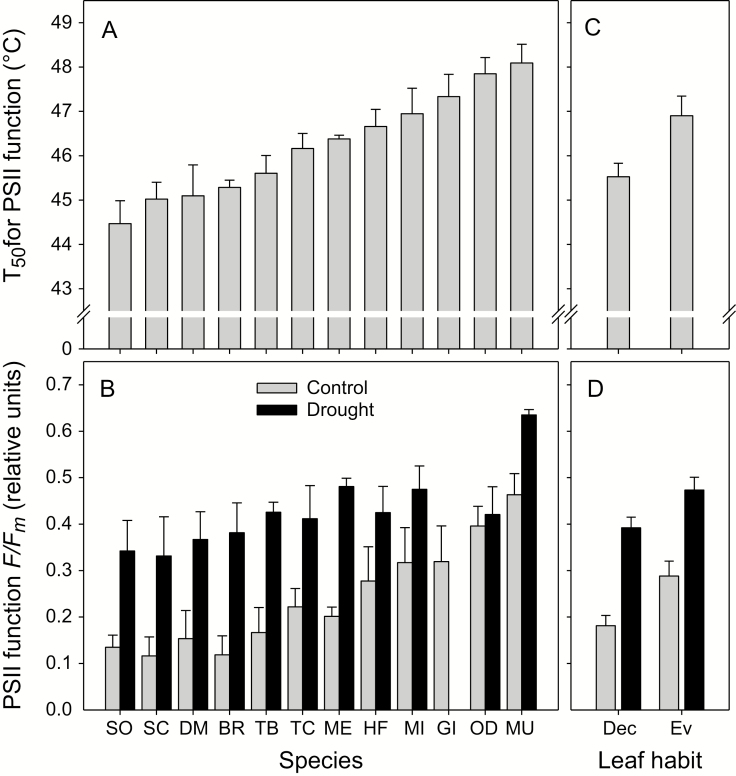
Thermotolerance in the study species. (A) T_50_ of PSII function in control (well watered) plants. (B) PSII function (*F*_*v*_   /*F*_*m*_) at 47.5 °C for control and drought-stressed plants. Error bars represent SE (*n* = 5–6). (C) and (D) Average thermotolerance for deciduous (Dec, *n* = 6) and evergreen (Ev, *n* = 6) leaf habits. Species names are provided in **Supporting Information—Table S1**.

Dark-adapted *F*_*v*_*   /F*_*m*_ ranged from around 0.7 to 0.8 at control temperature (25 °C) in all species, and this remained unchanged till temperatures increased above 40 °C (Fig. 1). There was a sharp decline in *F*_*v*_*   /F*_*m*_ after 40 °C, and at 50 °C this was reduced to negligible values for the more sensitive species, but not for the tolerant species. Estimates of T_50_ were significantly different between species and ranged from 44.5 °C in *Schleichera oleosa*, to 48.1 °C in *Memecylon umbellatum* (Fig. 2A; Table 1a). While not of primary interest we tested for differences between evergreen and deciduous leaf habits which showed higher T_50_ for evergreen than for the deciduous species (Fig. 2C; **Supporting Information—Table S3a**).

**Table 1.** (a) Variation in thermotolerance (T_50_ of PSII function, *F*_*v*_*   /F*_*m*_) for well-watered plants of the 12 study species. (b) Variation in thermotolerance (PSII function, *F*_*v   *_*/F*_*m*_ at 47.5 °C) for 11 species under control (well watered) and drought-stressed conditions.

Effect	df	MS	*F*	*P*
(a) Thermotolerance—T_50_ of PSII function (control plants)				
Species	11	7.39	7.61	<0.001
(b) PSII function at 47.5 °C (control vs. drought)				
Drought	1	1.26	74.13	<0.001
Species	10	0.1	5.88	<0.001
Drought × Species	10	0.009	0.51	0.879

At control temperatures (25 °C) there was no difference in dark-adapted *F*_*v*_*   /F*_*m*_ for plants that were drought stressed **[see Supporting Information—Fig. S3, Table S4]**. As with plants that were well watered, *F*_*v*_*   /F*_*m*_ decreased significantly at 47.5 °C, and was very low at 50 °C (Fig. 2B; also **see Supporting Information—Fig. S3**). However, drought-stressed plants performed significantly better than well-watered plants at these temperatures. The highest variation in *F*_*v*_*   /F*_*m*_ between species was observed at 47.5 °C, and we used *F*_*v*_*   /F*_*m*_ at 47.5 °C as a relative index of thermotolerance for the drought-stressed plants to test for differences between species, the effect of drought and the interactions between the two. Species differed significantly from each other, and drought resulted in better performance at this stressful temperature (Fig. 2B; Table 1b). The increase in thermotolerance in drought-stressed plants was similar across species. As seen with T_50_ for control plants, evergreen species had significantly higher *F*_*v*_*   /F*_*m*_ at 47.5 °C than deciduous species (Fig. 2D; **Supporting Information—Table S3b**).

The leaf traits measured, LMA, LDMC and LA differed significantly among species **[see Supporting Information—Fig. S4, Table S5]**. LMA and LA were not normally distributed and Kruskal–Wallis non-parametric analysis yielded similar results with significant differences between species for both traits. Photosynthetic rates varied nearly 2-fold from the least productive species to the most productive species under well-watered conditions species **[see Supporting Information—Fig. S5]**. Photosynthetic rates decreased sharply in drought-stressed plants, but the magnitude of decrease differed among species **[see Supporting Information—Fig. S5, Table S6]**. Average leaf wilting scores were significantly different across species and ranged from one in *M. umbellatum* indicating no wilting, to greater than three in *Bridelia retusa* indicating severe wilting and the beginning of leaf necrosis **[see Supporting Information—Fig. S5, Table S7]**. Leaf wilting scores were not normally distributed and Kruskal–Wallis non-parametric analysis yielded similar results with significant differences between species. Similarly, there was significant variation in leaf RWC which ranged from around 30 % to greater than 80 % **[see Supporting Information—Fig. S5, Table S7]**.

**Figure 3.**
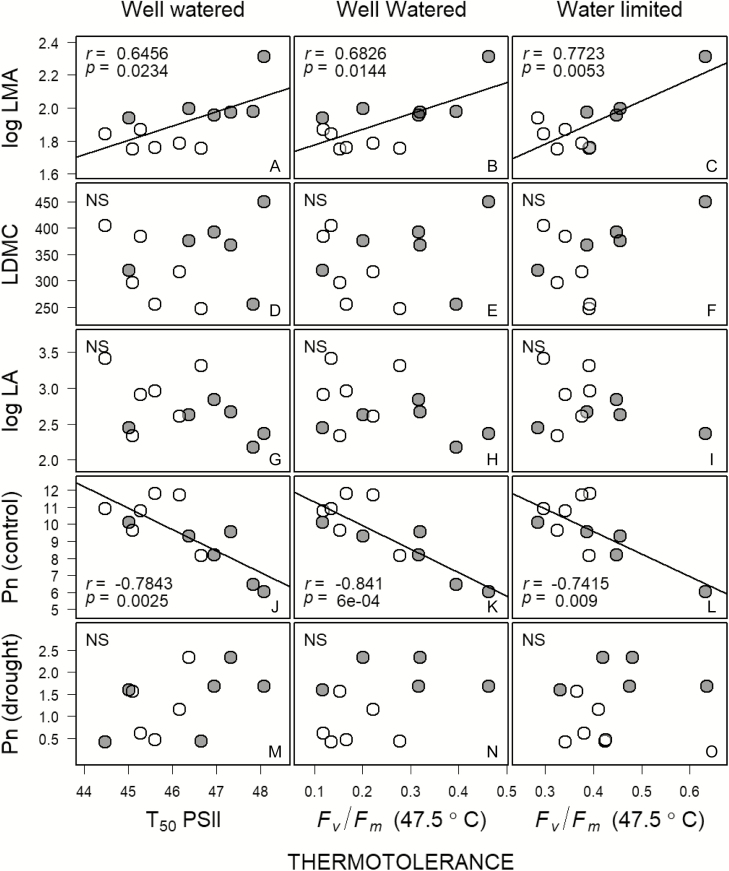
Relationship between thermotolerance and leaf traits. The open and closed gray circles represent deciduous and evergreen species, respectively. Thermotolerance for control plants (well watered) was quantified as T_50_ of PSII function (A, D, G, J, M), and *F*_*v*_   /*F*_*m*_ at 47.5 °C (B, E, H, K, N); and for drought-stressed plants (water limited) as *F*_*v*_   /*F*_*m*_ at 47.5 °C (C, F, I, L, O). LMA—leaf mass per area (g m^−2^), LA—leaf size (cm^2^), LDMC—leaf dry matter content (mg g^−1^), Pn—net photosynthesis (µmol m^−2^ s^−1^). LMA and LA were log-transformed to better approximate normality. Best fit lines were plotted using type II ordinary least squares linear regressions, ns—not significant at *P* < 0.05.

Thermotolerance for both control and drought-stressed plants as measured by T_50_ of *F*_*v*_*   /F*_*m*_, and *F*_*v*_*   /F*_*m*_ at 47.5 °C, respectively, was positively related to LMA, but not to LDMC or leaf size (Fig. 3). As LMA and LA were not normally distributed we examined Spearman’s rank correlations for these leaf traits with thermotolerance. Spearman’s rank correlations yielded significant positive relationships between LMA and T_50_ (control plants), but not for *F*_*v*_*   /F*_*m*_ at 47.5 °C (drought-stressed plants). Spearman’s rank correlations were not significant for either T_50_ of *F*_*v   *_*/F*_*m*_ in control plants, or *F*_*v*_*   /F*_*m*_ at 47.5 °C for the drought-stressed plants.

**Figure 4.**
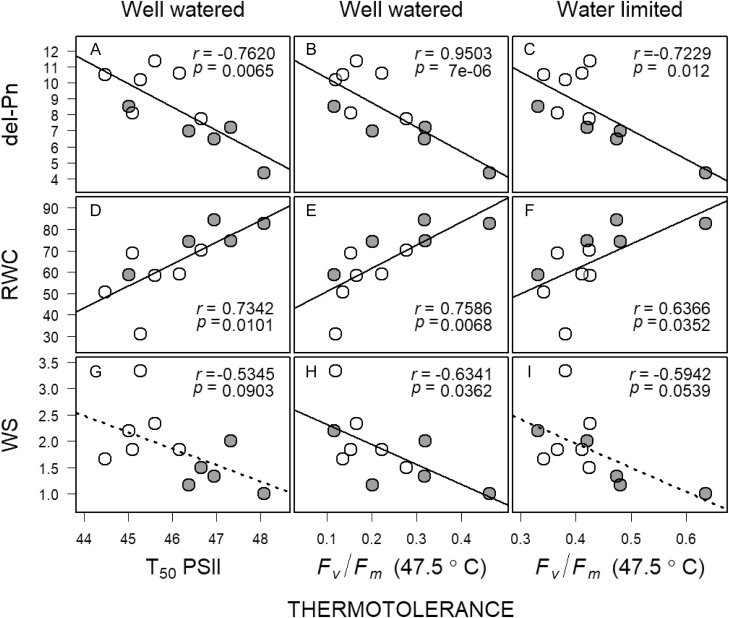
Relationship between thermotolerance and performance in drought-stressed plants. The open and closed gray circles represent deciduous and evergreen species, respectively. Thermotolerance for control plants (well watered) was quantified as T_50_ of PSII function (A, D, G), and *F*_*v*_   /*F*_*m*_ at 47.5 °C (B, E, H); and for drought-stressed plants (water limited) as *F*_*v*_   /*F*_*m*_ at 47.5 °C (C, F, I). Performance under drought stress was quantified as delPn—change in net photosynthesis after drought stress (µmol m^−2^ s^−1^), RWC—leaf relative water content (%) and WS—leaf wilting score (arbitrary units).

Thermotolerance (T_50_ of *F*_*v*_*   /F*_*m*_ in control plants, and *F*_*v*_*   /F*_*m*_ at 47.5 °C drought-stressed plants) was negatively related to photosynthetic rates for control plants, but not related to photosynthesis in the drought-stressed plants (Fig. 4). Finally, both measures of thermotolerance were significantly related to measures of drought tolerance, negative for change in photosynthesis with drought and wilting scores, and positive for leaf RWC (Fig. 4). The relationships with wilting scores were marginally significant (*P* < 0.1).

## Discussion

Leaf mass per area was positively related to thermotolerance in saplings of the 12 study species that were grown in a common environment. Congruent with this, thermotolerance was negatively related to photosynthetic rates. Species with higher LMA typically have greater leaf thickness, tissue density and greater investment in structural components, but lower nitrogen content and photosynthetic rates (Wright *et al.* 2004). The higher investment in LMA is associated with tougher leaves that are able to better withstand abiotic and biotic stress and sustain longer leaf longevity (Onoda *et al.* 2011). Our results suggest that the upper thermal limits of tolerance to high temperature in leaves vary along this “slow-fast” resource acquisition spectrum (Wright *et al.* 2004; Reich 2014).

**Figure 5.**
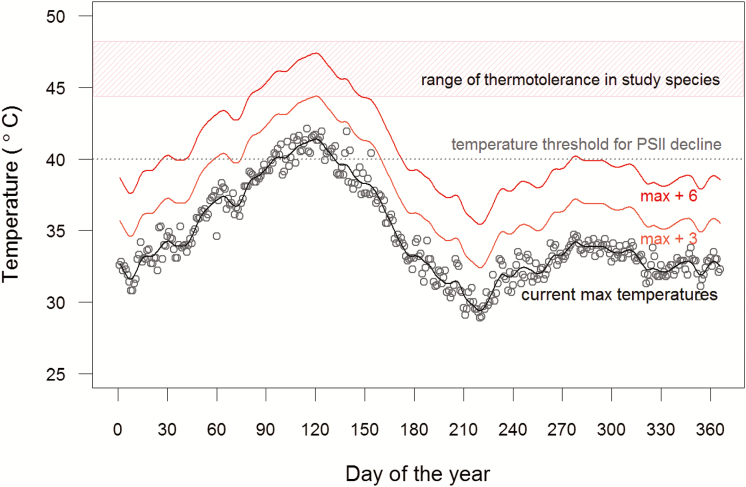
Maximum daily air temperatures in the region—data from Pune, India (2005–14). Absolute daily maximum temperatures are shown by the open circles, and the black line represents a smoothed curve fitted to this data (current max). We estimate future maximum temperatures by adding 3–6 °C to current maximum temperatures (Malhi *et al.* 2014)—this represents lower and upper predictions for the increase in temperatures in tropical regions by the end of the century (max + 3—orange line; max + 6—red line). The red hatched area highlights the range of thermotolerance observed in the studied species, and the grey dashed line represents the temperature above which we observed a significant decline in PSII function. Daily air temperature data were obtained from GHCN (Global Historical Climatology Network) daily Version 3.22.

Heat-induced damage and stress are likely to be ecologically relevant for these species in their natural habitat. Photosystem II function in all species declined sharply at temperatures higher than to 40 °C, and the temperatures that resulted in a 50 % loss of PSII function (T_50_), which represent temperatures that cause irreversible damage and necrosis (Bilger *et al.* 1984; Cunningham and Read 2006; Zhang *et al.* 2012) ranged between 44.5 and 48.1 °C. Daily maximum temperatures in this regions often exceed 40 °C (Fig. 5), and the highest maximum temperature of 42.1 °C recorded in the last decade is precariously close to the upper limits of thermotolerance for these species. Additionally, temperatures for sun-exposed leaves can be 5–15 °C higher than air temperatures when water availability and transpirational cooling are limited (Ishida *et al.* 1999; Vogel 2009; Krause *et al.* 2010; Schymanski *et al.* 2013). The hottest period in the year in this region comes at the end of the long dry season between April and May **[see Supporting Information—Fig. S1]**, when water availability is severely limiting. Finally, as in other dry tropical forests (Williams *et al.* 2008; Kushwaha *et al.* 2011) most species in this region are actively flushing new leaves between April and May, and recently flushed and immature leaves may be particularly vulnerable to heat stress and damage (Gauslaa 1984; Marias *et al.* 2017).

Future global warming and increased frequency of exposure to higher temperatures will likely have severe negative consequences for trees in this region. Temperatures in the tropics are predicted to increase by 3–6 °C over the course of this century (Malhi *et al.* 2014). A 3 °C increase in maximum temperatures will significantly increase the number of days when plants will experience temperatures greater the threshold for declines in PSII function (Fig. 5), and for the more sensitive species, like *S. oleosa* and *Diospyros montana*, this will result in exposure to temperatures near their upper limits of tolerance (T_50_). Future increases of 6 °C in maximum temperatures would exceed the T_50_ for all but the most tolerant species.

The observed relationship of thermotolerance with LMA and photosynthetic rates implies that species vulnerability to future climate change may not be random but related to functional attributes. This is particularly insightful because this allows us to extrapolate our results to other species beyond those examined in this study. Our results show that more productive species with lower LMA and higher photosynthesis are less thermotolerant, and therefore likely to be more vulnerable to future global warming, and this may result in directional changes in future species composition favouring species with high LMA and lower photosynthesis.

The LMA–thermotolerance relationship is congruent with predictions made by Curtis (2014), results from a previous field study of tropical tree species (Sastry and Barua 2017) and studies from other regions (Knight and Ackerly 2003; Gallagher 2014). However, Zhang *et al.* (2012) examined 24 woody species from a dry subtropical forest, and while they do not find any relationship with LMA, they report a significant positive relationship between thermotolerance and leaf longevity. Additionally, O’Sullivan *et al.* (2017) did not detect any relationship between thermotolerance and LMA in a global study of over 200 woody species. Thus, the observed relationships between leaf traits and thermotolerance may not be universal, but specific to certain environments, e.g. hot and arid environments. Given the predictive insight such relationships offer, especially in the context of understanding the responses of plants and communities to global warming, further studies in plants from diverse environments are needed to better appreciate the generality of leaf functional trait–thermotolerance relationships.

We observed higher average thermotolerance in evergreen than in deciduous species, as previously reported in tree species from this region (Sastry and Barua 2017). Leaves of evergreen species with longer leaf lifespans are likely to be exposed to a wider range of temperatures and higher extreme temperatures. This could explain the need for greater tolerance to extreme temperatures. Alternately, greater structural investment in leaves of evergreen species might result in greater tolerance to temperature extremes. These results suggest that fast-growing deciduous species may be more vulnerable to future warming, and this could lead to changes in the relative abundance of evergreen and deciduous species in these tropical dry forest communities.

Water limitation and drought resulted in the ability to withstand higher temperatures in these species that are adapted to hot and dry conditions. This has been documented in other plants (Havaux *et al.* 1988), including woody Mediterranean species from arid environments (Ladjal *et al.* 2000; Ghouil *et al.* 2003; Godoy *et al.* 2011). Such an increase in thermotolerance can result from the similar cellular effects, and crosstalk between the partially overlapping suite of cellular responses to drought and heat stress, including accumulation of stress proteins, anti-oxidants and reactive-oxygen species scavengers, and protective solutes (Wang *et al.* 2003; Pandey *et al.* 2015). Additionally, we observed that more thermotolerant species performed better when drought stressed as quantified by leaf wilting, leaf RWC and photosynthesis. Such a positive relationship between drought and temperature tolerance might be expected in plants adapted to hot and dry environments, and has been reported in varieties of cereals (Havaux *et al.* 1988), but to the best of our knowledge has not been documented in naturally occurring plants before. Understanding the nature of relationships between drought and high temperatures is likely to be very important for understanding the responses of plants to future climates where the likelihood of simultaneous exposure to extreme temperatures and reduced water availability are likely to increase (Niu *et al.* 2014).

Knowledge of the upper thermal limits of leaf physiology provides valuable insights into the relative heat sensitivity and potential vulnerability of plants to future warming and climate change. This is particularly true for trees where experimental exposure to high temperature for the whole organism over the entire lifetime of individuals is difficult. However, caution should be exercised in extrapolating these results to whole plant responses. Plant water use, leaf transpirational cooling, phenological and life history strategies, developmental and seasonal acclimation of thermotolerance to changing environmental conditions, etc. are likely to be important in determining the ultimate responses of trees to extreme climatic events. Future work that examines relationships between leaf thermotolerance and whole plant performance and survival is urgently needed to fully understand how forested communities will respond to future climate change.

## Conclusions

The upper limits of leaf thermotolerance in the saplings of the 12 study species were close to the maximum temperatures experienced in this region, and future increases in temperatures are likely to negatively impact most of these species. Exposure to drought increased thermotolerance, and across species, higher thermotolerance was positively related to greater drought tolerance. Notably, variation in thermotolerance was not random, but thermotolerance was higher in species with higher LMA and lower photosynthetic rates, and higher for evergreen than deciduous tree species. These differences in sensitivity to extreme temperatures imply differential vulnerability to future increases in extreme temperatures and drought which may favour directional changes in composition towards evergreen species with higher LMA and lower photosynthetic rates.

## Sources of Funding

This work was supported by intramural funding from IISER, Pune. A.S. was supported by a Senior Research Fellowship from the Council of Scientific and Industrial Research (CSIR), India.

## Contributions by the Authors

D.B., A.G. and A.S. conceived the study, designed the experiments, collected and analysed the data, and drafted the manuscript.

## Conflict of Interest

None declared.

## Supporting Information

The following additional information is available in the online version of this article—

**Table S1.** List of study species, family names, leaf habit and species codes used.

**Table S2.** Height and stem diameter of plants at the beginning of the experiment.

**Table S3.** Variation in thermotolerance between evergreen and deciduous leaf habit.

**Table S4.** Variation in thermotolerance (PSII function at 25, 47.5, 50 ŶC) for 11 species under control (well watered) and drought-stressed conditions.

**Table S5.** Variation in leaf functional traits for the 12 species: a) leaf mass per area (LMA); b) leaf dry matter content (LDMC); and c) leaf size (LA).

**Table S6.** Variation in photosynthetic rates for the 11 species under control (well watered) and drought conditions.

**Table S7.** Variation in drought performance in 11 species as evaluated by: a) leaf wilting stage scores; and b) leaf relative water content (RWC).

**Figure S1.** Climate data for the region from (Pune, Maharashtra, India).

**Figure S2.** Canopy fullness for study species from Bhimashankar Wildlife Sanctuary in the Northern Western Ghats, India.

**Figure S3.** The effect of drought stress on dark-adapted chlorophyll *a* fluorescence (*F*_*v*_/*F*_*m*_) at 25, 47.5 and 50 ŶC.

**Figure S4.** Leaf functional traits in the 12 study species: a) leaf mass per area; b) leaf dry matter content; c) leaf area.

**Figure S5.** Performance of the 12 species: a) photosynthesis under control and drought conditions; b) leaf wilting stage scores under drought conditions; c) leaf relative water content under drought conditions.

## Supplementary Material

Supporting Information
