# Supplementary material for: Leaf thermotolerance in dry tropical forest tree species: relationships with leaf traits and effects of drought
Source: AoB Plants. 2017 Dec 11;10(1):plx070. doi: 10.1093/aobpla/plx070 (PMC5767958; doi:10.1093/aobpla/plx070)
Supplement: Supporting Information [file plx070_suppl_supporting_information.docx]

**Supporting Information:**

### Leaf thermotolerance in dry tropical forest tree species: relationships with leaf traits and effects of drought

**Contents**

**Table S1:** List of study species, family names, leaf habit, and species codes used

**Table S2:** Height and stem diameter of plants at the beginning of the experiment.

**Table S3:** Variation in thermotolerance between evergreen and deciduous leaf habit.

**Table S4:** Variation in thermotolerance (PSII function at 25^o^C , 47.5^o^C , 50^o^C) for 11 species under control (well watered) and drought stressed conditions

**Table S5:** Variation in leaf functional traits for the 12 species: a) leaf mass per area (LMA); b) leaf dry matter content (LDMC); and, c) leaf size (LA)

**Table S6:** Variation in photosynthetic rates for the 11 species under control (well watered) and drought conditions

**Table S7:** Variation in drought performance in 11 species as evaluated by: a) leaf wilting stage scores; and, b) leaf relative water content (RWC)

**Figure S1:** Climate data for the region from (Pune, Maharashtra, India)

**Figure S2:** Canopy fullness for study species from Bhimashankar Wildlife Sanctuary in the Northern Western Ghats, India.

**Figure S3:** The effect of drought stress on dark adapted chlorophyll *a* fluorescence (*F_v_*/*F_m_*) at 25°C, 47.5°C, and 50°C

**Figure S4:** Leaf functional traits in the 12 study species: a) leaf mass per area; b) leaf dry matter content; c) leaf area

**Figure S5:** Performance of the 12 species: a) Photosynthesis under control and drought conditions; b) leaf wilting stage scores under drought conditions; c) leaf relative water content under drought conditions.

**Table S1:** List of study species, family names, leaf habit and species codes used.

| **Species** | **Family** | **Leaf habit** | **Code** |
| --- | --- | --- | --- |
| *Schleichera oleosa* (Lour) Merr. | Sapindaceae | Deciduous | SO |
| *Syzygium cumini* (L.) Skeels. | Myrtaceae | Evergreen | SC |
| *Diospyros montana* Roxb. | Ebenaceae | Deciduous | DM |
| *Bridelia retusa* (L.) A. Juss. | Phylanthaceae | Deciduous | BR |
| *Terminalia bellirica* (Gaertn.) Roxb. | Combretaceae | Deciduous | TB |
| *Terminalia chebula* Retz. | Combretaceae | Deciduous | TC |
| *Mimusops elengi* L. | Sapotaceae | Evergreen | ME |
| *Heterophragma quadriloculare*  (Roxb.) K. Schum. | Bignoniaceae | Deciduous | HF |
| *Mangifera indica* L. | Anacardiaceae | Evergreen | MI |
| *Olea dioica* Roxb. | Oleaceae | Evergreen | OD |
| *Garcinia indica (*Thouars) Choisy | Garcinieae | Evergreen | GI |
| *Memecylon umbellatum* Burm. F. | Melastomataceae | Evergreen | MU |

**Table S2:** Height and stem diameter of plants at the beginning of the experiment.

| Code | Species | n | height (cm) | SE | Diameter (mm) | SE |
| --- | --- | --- | --- | --- | --- | --- |
| BR | *Bridelia retusa* (L.) A. Juss. | 6 | 189.92 | 5.42 | 1.23 | 0.04 |
| DM | *Diospyros montana* Roxb. | 6 | 105.17 | 17.15 | 0.79 | 0.07 |
| GI | *Garcinia indica* (Thouars) Choisy | 6 | 45.50 | 4.25 | 0.41 | 0.04 |
| HF | *Heterophragma quadriloculare* | 6 | 52.62 | 2.01 | 1.13 | 0.07 |
|  | (Roxb.) K. Schum*.* |  |  |  |  |  |
| ME | *Mimusops elengi* L. | 6 | 96.18 | 4.05 | 0.87 | 0.02 |
| MI | *Mangifera indica* L. | 6 | 117.33 | 7.54 | 1.68 | 0.50 |
| MU | *Memecylon umbellatum* Burm. F. | 6 | 74.45 | 5.10 | 1.02 | 0.13 |
| OD | *Olea dioica* Roxb. | 6 | 104.67 | 11.67 | 0.98 | 0.15 |
| SC | *Syzygium cumini* (L.) Skeels. | 6 | 87.30 | 9.26 | 1.19 | 0.20 |
| SO | *Schleichera oleosa* (Lour) Merr. | 6 | 97.45 | 8.16 | 0.99 | 0.08 |
| TB | *Terminalia bellirica* (Gaertn.) Roxb. | 5 | 117.66 | 9.29 | 0.97 | 0.04 |
| TC | *Terminalia chebula* Retz. | 6 | 92.07 | 10.47 | 1.70 | 0.18 |

**Table S3:** Variation in thermotolerance between evergreen and deciduous leaf habit. a) For control (well watered) conditions - thermotolerance quantified as T_50_ of PSII function. Results shown are for an ANOVA with species nested within leaf habit. b) For control and drought stressed conditions - thermotolerance estimated as *F_v_/F_m_* at 47.5^o^C. Results shown are for an ANOVA with leaf habit (evergreen and deciduous) and drought treatment (control and drought) as fixed effects, and species nested within leaf habit.

| **Effect** | | ***df*** | | ***MS*** | ***F*** | | | ***p*** | |  | | |
| --- | --- | --- | --- | --- | --- | --- | --- | --- | --- | --- | --- | --- |
| **a) Thermotolerance - T_50_ of PSII function (control plants)** | | | | | | | | | | | | |
| Leaf Habit | | 1 | | 29.7 | 6.09 | | | < 0.05 | |  | | |
| Species (leaf habit) | | 10 | | 4.9 | 5.25 | | | < 0.001 | |  | | |
|  | |  |  | |  |  | |  | |  |  |  |
| **b) PSII function at 47.5°C (control vs. drought)** | | | | | | | | | | |  |  |
| Leaf Habit | | 1 | 0.252 | | 15.4 | < 0.001 | |  | |  |  |  |
| Species (leaf habit) | | 9 | 0.080 | | 4.9 | < 0.001 | |  | |  |  |  |
| Drought | | 1 | 1.228 | | 75.2 | < 0.001 | |  | |  |  |  |
| Drought x Leaf Habit | | 1 | 0.006 | | 0.4 | 0.541 | |  | |  |  |  |

**Table S4:** Variation in thermotolerance (PSII function, *F_v_/F_m_* at 25^o^C , 47.5^o^C , 50^o^C) for 11 species under control (well watered) and drought stressed conditions.

| **Effect** | ***df*** | ***MS*** | ***F*** | ***p*** |  |  |
| --- | --- | --- | --- | --- | --- | --- |
| Temperature | 2 | 12.74 | 1284 | <0.001 |  |  |
| Drought | 1 | 0.64 | 65 | <0.001 |  |  |
| Species | 10 | 0.11 | 12 | <0.001 |  |  |
| Temperature x Drought | 2 | 0.39 | 39 | <0.001 |  |  |
| Temperature x Species | 20 | 0.03 | 3 | <0.001 |  |  |
| Drought x Species | 10 | 0.01 | 1 | 0.624 |  |  |
| Temperature x drought x Species | 20 | 0.01 | 1 | 0.168 |  |  |

**Table S5:** Variation in leaf functional traits for the 12 species: a) leaf mass per area (LMA); b) leaf dry matter content (LDMC); and, c) leaf size (LA).

| **Leaf trait** | **Effect** | ***df*** | ***MS*** | ***F*** | ***p*** |  |  |
| --- | --- | --- | --- | --- | --- | --- | --- |
| a) LMA | Species | 11 | 9563 | 83.82 | < 0.01 |  |  |
| b) LDMC | Species | 11 | 2.51E+04 | 39.11 | < 0.01 |  |  |
| c) LA | Species | 11 | 3.58E+06 | 52.42 | < 0.01 |  |  |

**Table S6:** Variation in photosynthetic rates for the 11 species under control (well watered) and drought conditions.

| **Effect** | ***df*** | ***MS*** | ***F*** | ***p*** |  |  |
| --- | --- | --- | --- | --- | --- | --- |
| Drought | 10 | 7 | 4 | <0.001 |  |  |
| Species | 1 | 2183 | 1102 | <0.001 |  |  |
| Drought x species | 10 | 13 | 6 | <0.001 |  |  |

**Table S7:** Variation in drought performance in 11 species as evaluated by: a) leaf wilting stage scores; and, b) leaf relative water content (RWC).

|  | **Effect** | ***df*** | ***MS*** | ***F*** | ***p*** |  |  |
| --- | --- | --- | --- | --- | --- | --- | --- |
| a) Wilting stage | Species | 10 | 2.43 | 5.82 | < 0.01 |  |  |
| b) RWC | Species | 10 | 1391 | 10.09 | < 0.01 |  |  |

 **Figure S1:** Climate data for the region from (Pune, Maharashtra, India). a) Average daily minimum (green), maximum (dark red), and mean (black) air temperatures. Daily temperature data were averaged for a period of ten year prior to the study (2005-2014) and were obtained from GHCN (Global Historical Climatology Network) daily Version 3.22. b) Monthly averaged precipitation (1961-1990) - Dark grey vertical bars; and, sunshine duration (light grey curve). Precipitation and sunshine data were obtained from a high resolution global dataset (New *et al.* 2002).

**Figure S2:** Canopy fullness for study species from Bhimashankar Wildlife Sanctuary in the Northern Western Ghats, India. Data are from 15-30 individuals per species and were recorded monthly by visual estimation of canopy percentage between 2013-2015 (Barua unpubl. data). Species on the left are deciduous while those on the right are evergreen. Species names are provided in Table S1.

**Figure S3:** The effect of drought stress on dark adapted chlorophyll *a* fluorescence (*F_v_*/*F_m_*) at 25°C, 47.5°C, and 50°C. Black and grey bars represent control (well watered), and drought stressed conditions, respectively. Error bars represent standard error (n = 5 - 6). Species names are provided in Table S1.

**Figure S4:** Leaf functional traits in the 12 study species: a) leaf mass per area; b) leaf dry matter content; c) leaf area. Error bars represent standard error (n = 5 - 6). Species names are provided in Table S1.

**Figure S5:** Performance of the 12 species: a) Photosynthesis under control and drought conditions; b) leaf wilting stage scores under drought conditions; c) leaf relative water content under drought conditions. Error bars represent standard error (n = 5 - 6). Species names are provided in Table S1.
